# Pronator teres nerve branch transfer to the extensor carpi radialis brevis nerve branch for wrist extension reconstruction in proximal radial nerve injury following humeral shaft fractures

**DOI:** 10.1186/s12891-022-05950-1

**Published:** 2022-11-12

**Authors:** Jia Tian, Minghao Leng, Kun Wang, Qishun Huang

**Affiliations:** 1 Department of Hand Surgery, Union Hospital, Tongji Medical College, Huazhong University of Science and Technology, 1277 Jiefang Avenue, Wuhan, 430022 China; 2 Department of Orthopaedics, Suizhou Hospital, Hubei University of Medicine, Suizhou, 441300 China

**Keywords:** Nerve transfer, Tendon transfer, Proximal radial nerve injury, Humeral shaft fracture

## Abstract

**Background:**

Tendon and nerve transfers are used for functional reconstruction in cases of proximal radial nerve injury complicated by humeral fractures in patients who do not show functional recovery after primary nerve repair. The effectiveness of pronator teres (PT) nerve branch transfer to the extensor carpi radialis brevis (ERCB) nerve branch for wrist extension reconstruction was investigated and compared to the results of tendon transfer.

**Methods:**

This study included 10 patients with proximal radial nerve injury, who did not show functional recovery after primary nerve repair at our hospital between April 2016 and May 2019. The nerve transfer procedure included PT nerve branch transfer to the ECRB nerve branch to restore wrist extension and the flexor carpi radialis (FCR) nerve branch to the posterior interosseous nerve (PIN) to restore thumb and finger extension. Tendon transfer procedures included PT transfer to the ECRB for wrist extension, FCR transfer to the extensor digitorum communis (EDC) for finger extension and palmaris longus (PL) transfer to the extensor pollicis longus (EPL) for thumb extension.

**Results:**

Five patients recovered Medical Research Council grade M4 muscle strength in the ECRB and EPL in both tendon and nerve groups. Two patients recovered grade M3 strength and three patients recovered grade M4 strength in the EDC in the tendon transfer group, and all five patients recovered grade M4 strength in the EDC in the nerve transfer group. Limited wrist flexion was observed only in one patient in the tendon transfer group.

**Conclusion:**

PT nerve branch transfer to the ECRB nerve branch combined with FCR nerve branch transfer to PIN is a useful strategy for wrist and fingers extension reconstruction in patients with proximal radial nerve injuries.

**Supplementary Information:**

The online version contains supplementary material available at 10.1186/s12891-022-05950-1.

## Background

Proximal radial nerve injuries are commonly observed in clinical practice, with an incidence of approximately 22% in patients with humeral shaft fractures [1]. Tendon or nerve transfers are the main surgical options available for reconstruction of wrist, thumb, and finger extension in patients without recovery of function after primary repair. With regard to nerve transfers for wrist extension, the motor branch of the flexor digitorum superficialis (FDS) is commonly transferred to the motor branch of the extensor carpi radialis brevis (ECRB) for a synergistic effect [2, 3]. In patients in whom only one branch of the FDS nerve is identified, FDS flexion is compromised if that specific branch is cut off. In such cases, reportedly, pronator teres (PT) nerve transfer to the extensor carpi radialis longus (ECRL) nerve serves as a useful option to restore wrist extension [4]. However, the ECRB is universally preferred over the ECRL for wrist extension reconstruction [5–7], because its insertion is more central in location (into the 3rd metacarpal base) and therefore ensures better balanced radio-ulnar deviation during wrist extension. Meanwhile, the ECRB nerve branch is anatomically closer to the PT nerve branch and the suture can be done without tension. Therefore, we attempted PT nerve branch transfer to the ECRB nerve branch for wrist extension and compared our follow-up results with those of patients who underwent PT to ECRB tendon transfer. Correspondingly, flexor carpi radialis (FCR) nerve transfer to the posterior interosseous nerve (PIN) was performed to restore finger and thumb extension. In the tendon transfer group, the FCR was transferred to the extensor digitorum communis (EDC) to restore finger extension, and the palmaris longus (PL) was transferred to the extensor pollicis longus (EPL) to restore thumb extension and abduction.

## Methods

Between April 2016 and May 2019, 34 patients with proximal radial nerve injuries following humeral shaft fractures underwent conservative treatment or open primary repair of the radial nerve. The patients who achieved good functional recovery after intervention, or who had diabetes or psychiatric disorders which might significantly affect the surgical outcomes, were excluded. Ten patients who did not show functional recovery according to the electromyography after the first conservative treatment or open primary repair were selected for this study. They were divided into nerve transfer group and tendon transfer group based on the time they came back for follow-up check postoperatively. Five patients who presented within 9–12 months after primary repair underwent nerve transfer, and five patients who presented later than 12 months after primary repair underwent tendon transfer. Patients’ ages ranged between 26 and 46 years (mean 35.1 years). Eight patients with open wounds underwent open primary repair at the time of the first injury. The other two patients with closed humeral fractures primarily underwent conservative treatment. Both nerve and tendon transfers were performed by the same surgical team. Table 1 summarises the causes of injury, time of injury to surgery, and types of primary and secondary repair. All patients were evaluated using the Medical Research Council Grading System for muscle strength testing.

**Table 1:** Basic characteristics of the study group

Case No.	Gender	Age (years)	Cause	Type of primary repair	Time of injury to surgery (months)	Tendon/Nerve transfer
1	Man	32	Motorcycle	Direct suture	12	T
2	Woman	36	Car accident	Direct suture	9	N
3	Woman	26	Machine	Neurolysis	8	N
4	Man	45	Machine	Graft	14	T
5	Man	29	Falling	Nonoperative	13	T
6	Man	26	Motorcycle	Direct suture	10	N
7	Man	46	Machine	Direct suture	14	T
8	Woman	32	Bicycle	Nonoperative	9	N
9	Man	45	Falling	Direct suture	7	N
10	Man	22	Machine	Graft	15	T

### Tendon transfer procedure

**Fig. 1:**
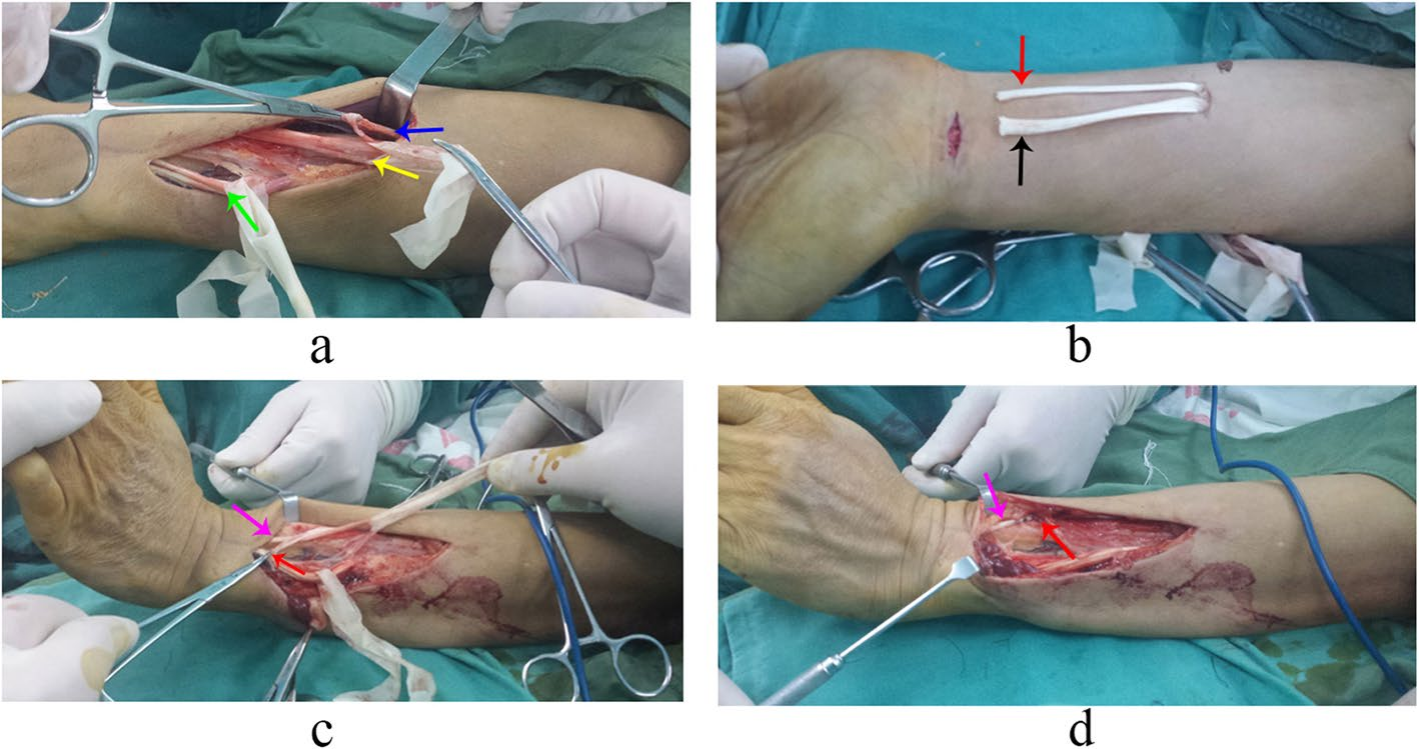
The surgical procedure of tendon transfer. **a** Pronator teres (PT) (blue arrow) was prepared. Extensor carpi radialis brevis (ECRB) (yellow arrow) was identified. Extensor digitorum communis (EDC) (green arrow) was identified. **b** Flexor carpi radialis (FCR) (black arrow) and Palmaris longus (PL) (red arrow) were sectioned from their ending points. **c** Palmaris longus (PL) (red arrow) was sectioned and pulled to the dorsal part of the forearm. Extensor pollicis longus (EPL) (purple arrow) was located. **d** Palmaris longus (PL) (red arrow) was sutured to extensor pollicis longus (EPL) (purple arrow)

The patient was placed in a supine position, and regional anaesthesia was administered to the affected extremity. Tendon transfers included PT to ECRB, FCR to EDC, and PL to EPL transfers. After the dorsal incision was made, a strip of periosteum from the radius, in continuity with the PT insertion was separated and prepared (Fig. 1a). The PT was freed in the proximal direction to achieve maximum excursion. The ECRB and EDC tendons were identified and labeled with a rubber band (Fig. 1a). Then, another incision was made in the volar side, where the FCR and PL tendons were dissected (Fig. 1b) and freed as proximal to the muscle belly as possible through the subcutaneous tunnel. The EPL was rerouted along the radial border of the thumb metacarpal (Fig. 1c) and coapted with the PL to restore thumb extension (Fig. 1d), with the wrist in a neutral position and maximum tension on both the PL and the distal stump of the EPL. The FCR was passed subcutaneously around the radial border of the forearm. The EDC was divided just proximal to the retinaculum and coapted with the sectioned end of the FCR with the wrist and metacarpophalangeal joints in a neutral position and the FCR under maximum tension. The PT was subsequently woven around the radial border of the forearm and transferred to the ECRB distal to the musculotendinous junction, with the wrist at 45° of extension and the PT under maximum tension. The degree of finger extension was carefully checked after the tendon was sutured. We confirmed that no patient had any abnormal extension of the fingers, which requires appropriate adjustment of tension in the corresponding tendon. Pulvertaft weave method was used to suture the donor and recipient tendons. The tourniquet was used to facilitate the dissection procedure and released within 40 min. The arm was immobilised in a cast, with the elbow in 90° flexion, the forearm pronated, the wrist in 50° extension, the thumb extended and abducted, and the fingers in a resting position.

### Rehabilitation

The patient’s upper limb was placed in a long-arm cast in the position described above. The cast lasted 3 weeks, then a short splint was used with the elbow free, the wrist in 30 degrees of extension, and metacarpophalangeal (MCP) joints at neutral. Assisted active and passive exercises were initiated. The splint was discontinued 6 weeks after surgery. Then the patient was transitioned to night splinting only. Exercises were performed in a sequence: flexion-extension of the elbow with the wrist and fingers in extension, flexion-extension of the wrist with the fingers extended and the elbow flexed, and flexion of the MCP joints with the wrist extended and the elbow flexed. Eight to 10 weeks after surgery splinting was discontinued and strengthening was commenced. Unrestricted activity was allowed after 12 weeks.

### Nerve transfer procedure

**Fig. 2:**
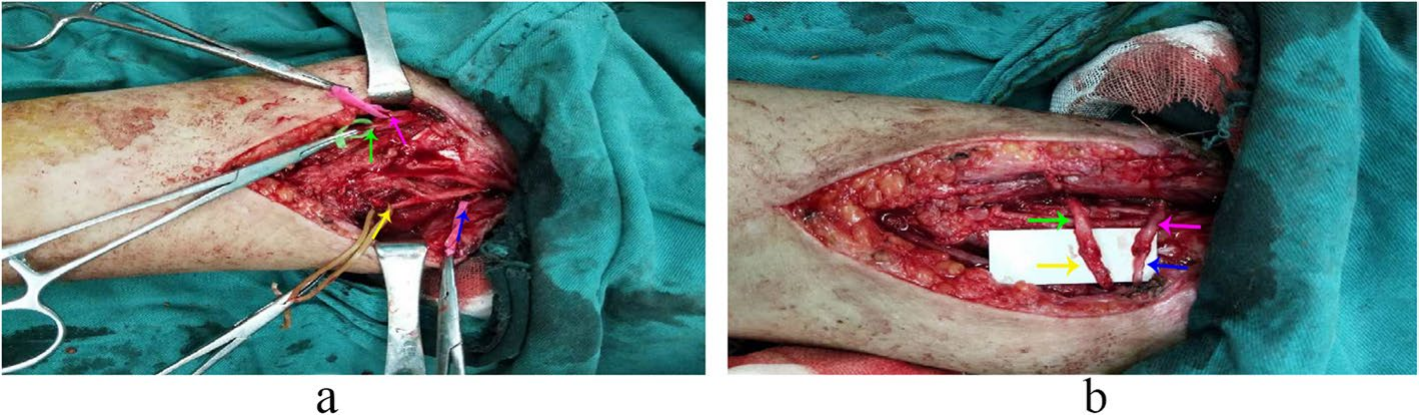
The surgical procedure of nerve transfer. **a** FCR motor nerve branch (yellow arrow) and PT motor nerve branch (blue arrow) were exposed. ECRB motor nerve branch (purple arrow) and PIN (green arrow) were identified. **b** PT motor nerve branch (blue arrow) was anastomosed to ECRB motor nerve branch (purple arrow). FCR motor nerve branch (yellow arrow) was anastomosed to PIN (green arrow)

The patient was placed in a supine position, and general anaesthesia was administered. Muscle relaxants were not used intraoperatively. We created an incision from the mid forearm to the lateral antecubital fossa. The median nerve was carefully dissected. The FCR and PT nerve branches were exposed and (Fig. 2a) confirmed using intraoperative electrical stimulation. The ECRB nerve branch and the PIN were identified along the course of the radial nerve (Fig. 2a). The PT nerve branch was sectioned distally and anastomosed to the ECRB nerve branch (Fig. 2b), which was cut at the branching point from the median nerve. Following careful proximal dissection, the FCR nerve branch was dissected distally and anastomosed to the PIN (Fig. 2b). Under microscopic guidance, we performed end-to-end suturing of the sectioned nerve ends. The tourniquet was used to guarantee a quick identification of the nerve branches by intraoperative electrical stimulation, and released within 30 min. The arm was immobilised in a splint with elbow in 90° flexion, forearm in pronation, wrist in neutral position, and fingers free. Each patient took Mecobalamin tablets after meal (0.5 mg/time, 3 times/day) for 1 month.

### Rehabilitation

In the first 2 weeks, splint was used to protect the nerve coaptation site. The sutures and the splint were removed after the first 2 weeks, and donor activation exercises including resisted finger flexion and isometric wrist flexion were initiated. From 3 weeks to 4 months postoperatively, exercises were commenced to pair the donor movement with the recipient movements through active donor movement and assisted recipient actions. This is important to reinvervate recipient muscle by contracting the donor muscle. Moreover, electrical stimulation were commenced after 6 weeks. From 3 months to 12 months, muscle strengthening was performed by activating the recipient muscles with or without blocking the donor action. Focused motor reeducation was integrated in all physical therapy sessions.

## Results

Table 2 shows differences between the tendon and nerve transfer groups, 3 years postoperatively. Videos 1 and 2 demonstrate the recovery of the extension function of wrist and fingers at the 3-year follow-up who underwent tendon/nerve transfer separately.

**Table 2:** The follow-up results of patients in tendon/nerve transfer group

Patient number	Muscle strength (Pre/Post Operation)			Wrist flexion limitation	Extending fingers independently	DASH score	Time to reach the final results (months)
	ECRB	EDC	EPL				
1	M0/M4	M0/M3	M0/M4	Yes	No	18	4
2	M0/M4	M0/M4	M0/M4	No	Yes	9	10
3	M0/M4	M0/M4	M0/M4	No	Yes	10	8
4	M0/M4	M0/M3	M0/M4	No	No	21	5
5	M0/M4	M0/M4	M0/M4	No	No	9	5
6	M0/M4	M0/M4	M0/M4	No	Yes	14	9
7	M0/M4	M0/M4	M0/M4	No	No	10	6
8	M0/M4	M0/M4	M0/M4	No	Yes	12	11
9	M0/M4	M0/M4	M0/M4	No	Yes	15	10
10	M0/M4	M0/M4	M0/M4	No	No	9	4

*ECRB* Extensor Carpi Radialis Brevis, *EDC* Extensor Digitorum Communis, *EPL* Extensor Pollicis Longus

### Tendon transfer group

Five patients recovered M4 grade muscle strength in the ECRB. Two patients recovered grade M3 strength, and three patients recovered grade M4 strength in the EDC. All five patients recovered grade M4 strength in the EPL. One patient showed limited wrist flexion. Independent finger extension was limited in all patients. Four patients developed muscle fatigue after continued movement. No patient showed clear loss of pronation strength. The mean Disabilities of Arm, Shoulder, and Hand (DASH) score was 13.4 (range 9–21).

### Nerve transfer group

All five patients recovered grade M4 muscle strength in the ECRB, EDC, and EPL. No patient showed restricted wrist flexion. Independent finger extension was observed in all patients, without significantly reduced strength in individual fingers. No patient developed muscle fatigue after continued movement, and no patient showed clear evidence of loss of pronation strength. The mean DASH score was 12 (range 9–15).

## Discussion

Surgery is warranted to restore wrist and finger extension in patients who do not show signs of spontaneous recovery 6–9 months after radial nerve injury. The most common tendon transfer combinations for radial nerve palsy include PT to the ECRB, FCR to the EDC, and PL to the EPL [8]. PT transfer to the ECRB is commonly used for wrist extension reconstruction, and the results are satisfactory in most cases. Surgeons should be mindful that PT transfer to the ECRB should necessarily include a strip of periosteum from the radius in continuity with the PT insertion to ensure PT tendon tissue of sufficient length, which can be conveniently sutured with the ECRB using a weave pattern. Notably, median nerve branch transfer is an increasingly popular approach used in recent years for wrist extension restoration. Several studies have reported the use of the FDS motor nerve branch as a donor branch transferred to the ECRB motor branch [2, 3]; this method is in agreement with the fundamental principle of tendon transfer, which emphasises the synergistic relationship between finger flexion and wrist extension to reduce the motor re-education time [9]. However, this approach cannot be used in patients with only one nerve branch to the FDS. Caetano et al. reported that 44% of cadavers showed only one branch to the FDS from the median nerve [10]. Garcia-Lopez et al. proposed the use of the PT nerve branch as a donor nerve transferred to the ECRL nerve branch [4]. The ECRL nerve branch was selected as a recipient considering the wide variability in the origin of the ECRB branches and the possibility of misidentification of the ECRB branch with that to the supinator [4]. However, the motor branch to ECRB can be easily identified closed to the cutaneous branch of the radial nerve, parallel to it and under the brachioradialis muscle. Conversely, the two branches of the supinator muscle lie parallel to the posterior interosseous nerve and take another way going into the supinator muscle. Significant difficulty as observed by García-López was not encountered by us during PT nerve branch transfer to the ECRB nerve branch and excellent wrist extension function was achieved in our patients. On the other hand, sometimes there are 2 different motor branches to PT, sometimes one motor branch comes from the median nerve together with FCR/PL motor branch [11, 12]. If there are two nerve branches to PT, we usually choose the longer one to make sure the suture without any tension. If there is only one motor branch arising from the median nerve together with FCR/PL motor branch, we will preserve the nerve fascicles to FCR/PL by intraneural dissection from the bifurcation point to the median nerve. The number of branches to the PT may not be important because the biceps and pronator quadratus muscles compensate for loss of function of the PT. Therefore, using the PT as a donor nerve transferred to the ECRB nerve branch is a safe and feasible strategy for restoration of wrist extension.

In view of the synergistic relationship between finger extension and wrist flexion, the FCR is frequently transferred to the EDC for finger extension reconstruction. Although transfer of the FCU tendon for finger extension can provide better strength respect to FCR tendon transfer [13], interference with physiological radial deviation during wrist extension and ulnar deviation during wrist flexion is a common complication after surgery. Several studies recommend changes in the alignment of the FCU to prevent radial deviation after wrist extension reconstruction [6]. Fortunately, the FCR motor nerve branch can be transferred to the PIN to restore finger extension without this problem [3]. Sallam et al. observed good recovery of finger and thumb extension after transfer of both the FCR and PL motor branches to the PIN [9]. All patients who underwent successful nerve transfers could perform finger extension with the wrist in an extended or neutral position. However, nearly 50% of patients in the tendon transfer group had to perform full wrist flexion to achieve full finger extension [14].

The PL is often transferred to the EPL for thumb extension reconstruction; the EPL is usually transected at its musculotendinous junction and is rerouted to pass along the radial border of the thumb metacarpal. The distal end of the PL is sutured to the proximal end of the EPL by rerouting the EPL tendon in the 1st or 2nd extensor compartment of the wrist. These tips and tricks are useful to obtain a better first ray opening of the thumb in abduction. Unfortunately, most patients show an extension lag at the metacarpal joint during follow-up because lack of abductor pollicis longus (APL) and extensor pollicis brevis (EPB) tendon reanimation. This phenomenon is rare in nerve transfer for PIN because EPL, APL and EPB can be reanimated obtaining better first ray opening in abduction. Meanwhile, there are 2 main fascicles in PIN: one is for the thumb (EPL, APL, EPB) and extensor indicis proprius (EIP), one is for the EDC, extensor digiti minimi (EDM) and extensor carpi ulnaris (ECU). The suture of a single motor branch (of the FCR or FDS) to the PIN can present a mismatch. Therefore, a few authors recommend additional transfer of the distal anterior interosseous nerve to the deep branch of the PIN that innervates the thumb extensor muscles for full recovery of thumb extension [15]. A good match between the donor and recipient nerve endings is important to ensure favourable recovery [16].

Tendon suture techniques, postoperative rehabilitation, possibility of spontaneous recovery, and nutraceuticals are among the factors that affect final outcomes. Suturing techniques used in tendon transfer include the Pulvertaft weave, side-to-side, spiral weave, lasso, and double loop [17–19]. With regard to biomechanical strength, the side-to-side method is widely preferred over the Pulvertaft weave as a simple method to adjust tension [20, 21]. Early postoperative rehabilitation significantly affects final functional outcomes. However, some authors have a different opinion. Altintas et al. [22] reported favourable outcomes in a series of patients who underwent tendon transfer but did not receive postoperative hand therapy. Studies have reported a high rate of spontaneous recovery in patients with radial nerve paralysis following humeral fractures [23, 24]. Reinnervation of the brachioradialis and ECRL is considered a clear indication of spontaneous recovery according to the electromyography results. Spontaneous recovery is usually ruled out for > 6 months after the injury, after which it is unlikely to occur. All patients in a group underwent the same surgical procedure and rehabilitation program to minimise intragroup bias. In recent years, there has been an increasing number of articles published on the role of nutrients in nerve regeneration [25–28]. Therefore, different dietary habits may also be a factor that can not be neglected when treating patients with nerve injury. A variety of foods with different combinations of nutrients that are likely to have synergistic effects could differ in the effect on the nerve regeneration. It is a big challenge to make sure patient compliance in dietary guidance after surgery. Our patients are advised to take normal food just as they eat in their daily life except for Mecobalamin tablets, which may affect recovery outcomes due to the different dietary habits.

The limitations of this study include a selection bias and a short follow-up period. Patient selection for nerve or tendon transfers was primarily based on the time at which they were admitted to our department. Patients were not randomly grouped, which may have led to a selection bias. Moreover, the length of postoperative follow-up was insufficient. Further studies with long-term follow-up are warranted to identify more unexpected complications.

## Conclusions

PT nerve branch transfer to the ECRB nerve branch combined with FCR nerve branch transfer to the PIN is a useful strategy for wrist and fingers extension reconstruction in patients with proximal radial nerve injuries.

## Supplementary Information

**Additional file 1: Video 1.** The patient who underwent tendon transfer in Fig. 1 recovered his extension function of wrist and fingers at the 3-year follow-up.

**Additional file 2: Video 2.** The patient who underwent nerve transfer in Fig. 2 recovered his extension function of wrist and fingers at the 3-year follow-up.

## Data Availability

All data generated or analysed during this study are included in this published article.

## Abbreviations

PT: Pronator teres
ERCB: Extensor carpi radialis brevis
FCR: Flexor carpi radialis
PIN: Posterior interosseous nerve
EDC: Extensor digitorum communis
PL: Palmaris longus
EPL: Extensor pollicis longus
EPB: Extensor pollicis brevis
APL: Abductor pollicis longus
ERCL: Extensor carpi radialis longus
FCU: Flexor carpi ulnaris
EDM: Extensor digiti minimi
EIP: Extensor indicis proprius
ECU: Extensor carpi ulnaris
